# BHX Inhibits the Wnt Signaling Pathway by Suppressing β-catenin Transcription in the Nucleus

**DOI:** 10.1038/srep38331

**Published:** 2016-12-02

**Authors:** Fengxia Ding, Meisa Wang, Yibo Du, Shuangshuang Du, Zhongling Zhu, Zhao Yan

**Affiliations:** 1 Department of Clinical Pharmacology, Tianjin Medical University Cancer Institute and Hospital, National Clinical Research Center for Cancer, Key Laboratory of Cancer Prevention and Therapy, Tianjin 300060, P. R. China

## Abstract

BHX (N-(4-hydroxybenzyl)-1,3,4-triphenyl-4,5-dihydro-1H-pyrazole-5-carboxamide), a Wnt signaling pathway inhibitor, effectively inhibits tumor cell growth, but the underlying mechanism is unclear. Thus, we aim to investigate the effects and associated mechanism of BHX action on A549 and MCF-7 cell lines. In our study, MTT(3-[4,5-dimethyl-2-thiazolyl]-2,5-diphenyl-2H-tetrazolium bromide) and xenograft model assay indicated that cell growth was inhibited by BHX at a range of concentrations *in vitro* and *in vivo*. The expression of β-catenin and Wnt signaling pathway downstream target genes were decreased evidently under BHX treatment. Flow cytometry also revealed that BHX treatment significantly induced G1 arrest. Further analysis showed that BHX lowered the transcriptional level of β-catenin. In conclusion, BHX inhibited the nuclear synthesis of β-catenin, thereby suppressing the Wnt signaling pathway and further inhibiting tumor growth and proliferation.

Cancer is a condition that results from the joint involvement of multiple genes, factors, and stages. Cellular signal transduction pathways play a vital role in tumor development and occurrence^1^. The Wnt signaling pathway is highly involved in cell proliferation, migration, apoptosis, and fate determination during early embryonic growth and development^2^,^3^. At present, the Wnt/β-catenin signaling pathway is an intensively studied signal transduction pathway, which is also highly conserved throughout evolution^4^,^5^. Aberrant Wnt/β-catenin pathway activation is strongly linked to the development of many cancers^6^,^7^. When the canonical Wnt signaling pathway is activated abnormally, the Axin/GSK-3/APC complex cannot phosphorylate β-catenin, leading to the latter’s ubiquitination and subsequent degradation. Non-phosphorylated β-catenin accumulates in the cytoplasm, so that cytoplasmic β-catenin is stabilized and transported further into the nucleus and interacts with TCF/LEF^3^,^8^. Subsequently, the Wnt pathway downstream target genes are involved in cell fate and proliferation regulation. These target genes include c-myc, cyclin D1, c-Jun, c-fos, and some members of the Fra-1 and AP-1 family^9^,^10^.

Wnt signaling pathway includes a variety of proteins, abnormal transcription and expression of signaling molecules are the main reason that activate Wnt signaling pathway^11^,^12^. Therefore, various specific targeted drugs have been developed. Inhibitors that disrupt the β-catenin and TCF interaction have been widely explored, as well as RNAi approaches, ICG-001, PKF115–584^13^,^14^. There are also some inhibitors targeting Frizzled such as OMP-18R5 and 3289–8625^15^. However, toxicity, side effects and other shortcomings appear. In spite of these potential hurdles, research toward identifying potent Wnt pathway antagonists for cancer treatment is promising.

On the basis of previous studies, we generated a computer-aided design of small-molecule inhibitors and synthesized the novel pyrazoline derivative BHX (N-(4-hydroxybenzyl)-1,3,4-triphenyl-4,5-dihydro-1H-pyrazole-5-carboxamide) to block Wnt signaling^16^. We discovered that BHX could inhibit the growth of cancer cell, but the mechanism was unclear. In this study, we evaluated the anti-cancer effect of BHX on lung and breast cancers and then discovered the mechanisms of the underlying effects. Our results provide a rationale for the further development of BHX as a prospective therapeutic agent for cancer that involves Wnt pathway activation.

## Results

### BHX inhibits the proliferation of cancer cell *in vitro* and *in vivo*

**Figure 1:**
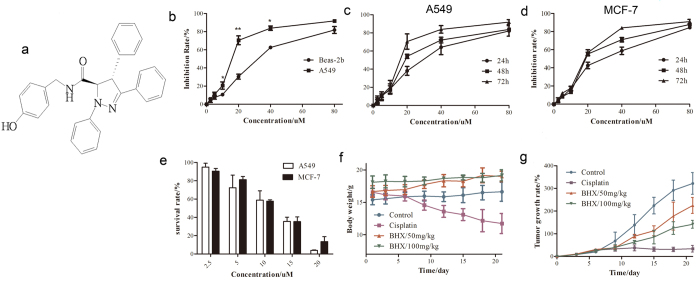
Antiproliferative effects of BHX on tumor and normal lung epithelial cells. (**a**) Chemical structure of BHX. (**b**) Beas-2b and A549 cells were treated with different concentrations (0–80 μM) of BHX for 72 h. Cell viability was analyzed by MTT assays. (**c**,**d**) A549 and MCF-7 cells were treated with different concentrations (0–80 μM) of BHX for different times. (**e**) Colony-formation assays of the MCF-7 and A549 cell treated with BHX at an indicated concentration. Results are mean ± SD (n = 3). *p < 0.05, **p < 0.01. Tumor-bearing mice were treated intraperitoneally with vehicle, BHX (50 and 100 mg/kg), and cisplatin for 21 days. The body weight (**f**) and tumor growth rate (**g**) were measured as described in the Materials and Methods section.

To explore the tumor inhibitory effect of BHX *in vitro* and *in vivo*,We first examined the cytotoxicity of BHX in human lung cancer cell lines and normal lung epithelial cells (Beas-2b). BHX(molecular structure were shown in Fig. 1a) displayed moderate cytotoxicity to the Beas-2b cells with an IC50 of 28.08 μM by MTT assay. However, the IC50 of the A549 cells was 15.49 μM (Fig. 1b), which was significantly lower than that of the Beas-2b (Fig. 1c). We selected another Wnt-pathway-activated cell, i.e., the breast cancer cell line MCF-7. BHX inhibited tumor cell proliferation in a dose-dependent manner by the MTT assay (Fig. 1d). BHX also suppressed the clonogenic activity of these two cell lines (Fig. 1e).

Then, we used BALB/c nude mice, which were implanted with A549 cells by subcutaneous injection, for testing against cisplatin as positive control^17^, In the cisplatin group, the growth of the implanted tumors was significantly inhibited, but the weight of the cisplatin group decreased rapidly, and two nude mice died. However, the weights of the nude mice in the control and BHX groups did not change (Fig. 1f). At the same time, BHX inhibited tumor growth significantly. The tumor growth inhibition of the high-dose group was stronger than that of the low-dose group but lower than that of the cisplatin group (Fig. 1g). Although cisplatin showed a strong tumor inhibitory effect in the A549 tumor-bearing mice, the drug exhibited substantial toxicity, unlike BHX, which showed almost no toxicity. All the results proved that BHX effectively inhibited human tumor cell proliferation. Moreover, BHX showed lower toxicity effect *in vitro* and *in vivo*.

### BHX reduces endogenous β-catenin and decreases its translocation into the nucleus

**Figure 2:**
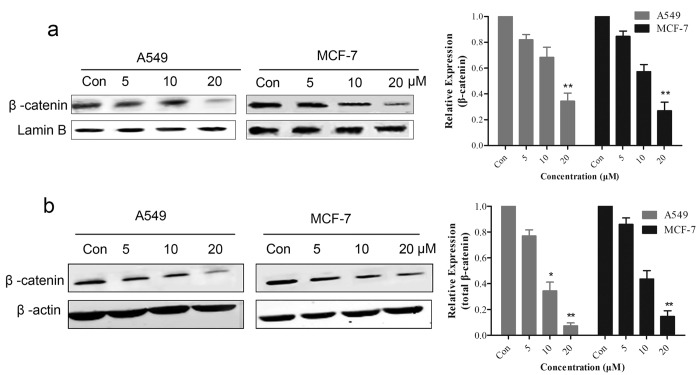
Effect of BHX on the nuclear expression of β-catenin, the total β-catenin protein levels in A549 and MCF-7 cells. (**a**) Cell nuclear protein extracts prepared from tumor cell treated with or without BHX (0–20 μM) for 72 h examined by Western blot. Lamin B served as the internal control in the nucleus. BHX did not affect Lamin B expression. After treatment with BHX, the expression level of β-catenin in the nucleus significantly decreased. (**b**) Expression of the β-catenin protein in the A549 and MCF-7 cells treated with or without (0–30 μM) BHX and β-actin served as an internal control. BHX treatment did not affect β-actin expression, whereas the total β-catenin expression was significantly decreased. Results are mean ± SD (n = 3). *p < 0.05, **p < 0.01.

To determine whether the tumor cell growth inhibitor effect of BHX in breast and lung cancer cells is mediated by the regulation of the Wnt/β-catenin signaling components, we carried out Western blot assay. The expression of the β-catenin protein was also diminished significantly following the 72 h BHX treatment. After BHX treatment, the expression levels of β-catenin in the nucleus were decreased (Fig. 2a). We also examined the expression levels of total β-catenin in A549 and MCF-7 cells by Western blot. Findings clearly showed that the expression of total β-catenin also decreased compared with the control (Fig. 2b).

**Figure 3:**
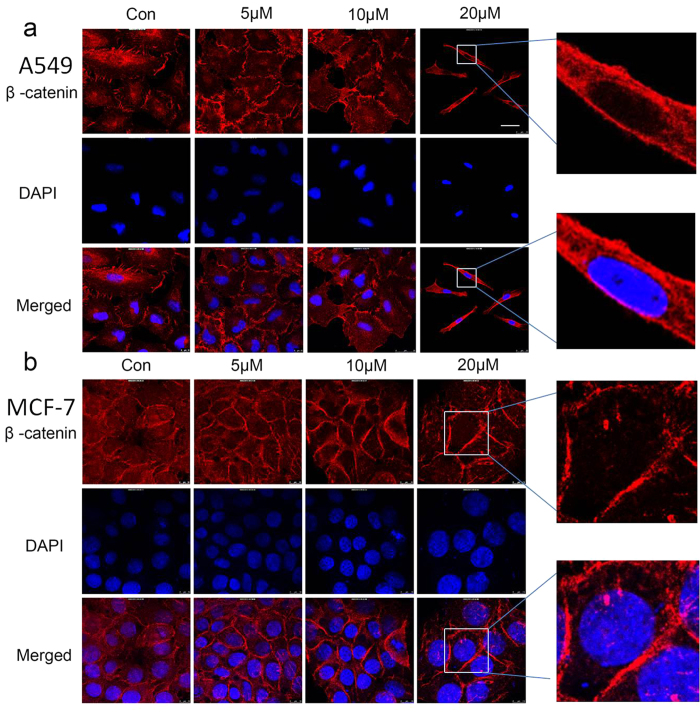
Effect of BHX on the localization in A549 (**a**) and MCF-7 (**b**) cells. Localization of β-catenin in cells treated with or without BHX (0–20 μM) for 72 h by immunofluorescence. A high level of β-catenin staining (red) is displayed in the nucleus of the untreated cells. However, in the cells treated with BHX, a small amount of β-catenin is shown in the nucleus, and even absent in some cell nucleus. Scale bar in a, same for b (expect the pictures which are magnified): 15 μm.

The nuclear translocation of β-catenin is a crucial factor in the activation of the Wnt/β-catenin signaling pathway. Then we checked the effect of BHX on the location of nuclear β-catenin by immunofluorescence assay. Results revealed that β-catenin levels were decreased in the cell nucleus (Fig. 3a,b).

### BHX regulates the cell cycle by controlling the Wnt signaling pathway

**Figure 4:**
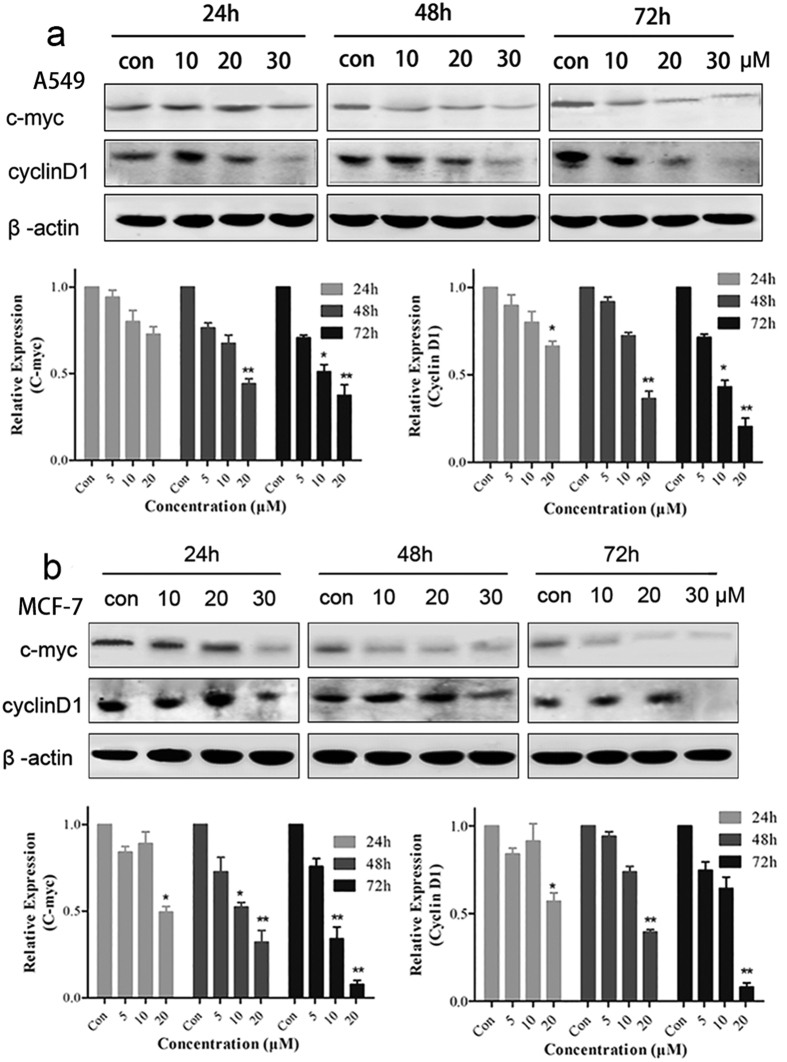
BHX-induced down-regulation of cyclin D1 and c-myc protein expression. A549 (**a**) and MCF-7 (**b**) cells were incubated with various concentrations (0–30 μM) of BHX for different time periods. Cyclin D1 and c-myc protein expression levels were detected by Western blot in which β-actin served as internal control. The relative cyclin D1 and c-myc protein expression levels were quantified by Image J. Results are mean ± SD (n = 3). *p < 0.05, **p < 0.01.

When the Wnt signaling pathway is activated, β-catenin interacts with the TCF and lymphoid enhancer–binding factor (LEF) in the nucleus. This interaction activates downstream target genes transcription, including those of c-Myc and cyclin D1, which are involved in the regulation of cell proliferation and cell cycle^18^,^19^. To further confirm the effect of BHX on the Wnt signaling pathway, we analyzed the Wnt pathway downstream target genes c-myc and cyclin D1 by Western blot. Cyclin D1 is essential for the G1/S transition in the cell cycle. Western blot analysis showed that the BHX treatment markedly reduced the expression of cyclin D1 and c-myc in the tumor cells (Fig. 4a,b).

**Figure 5:**
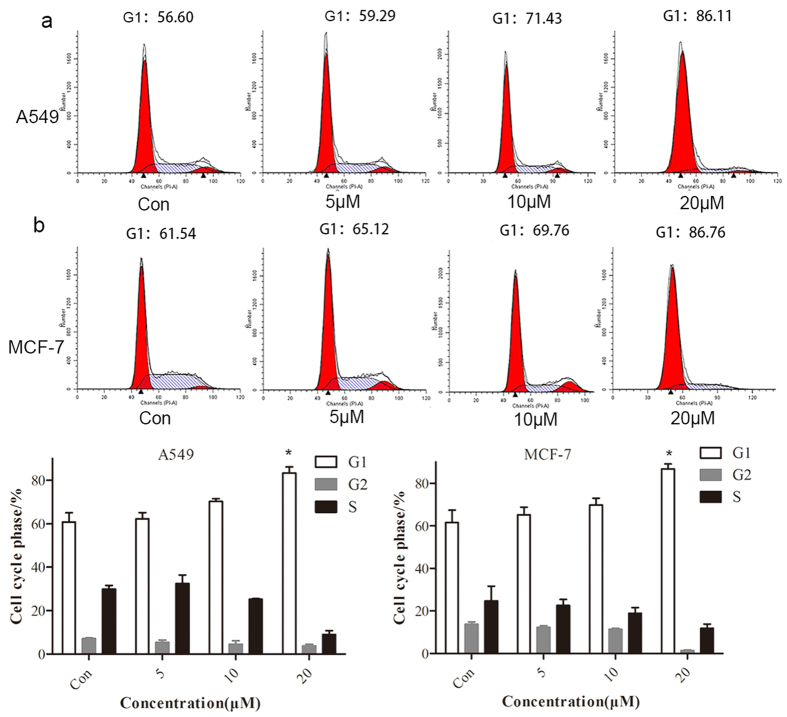
BHX-triggered cell-cycle arrest at the G1/S phase in the A549 and MCF-7 cells. A549 (**a**) and MCF-7 (**b**) cells treated with various concentrations of BHX for 48 h. We analyzed the cell-cycle distribution using Verity’s Modfit LT 3.0 software. Results are mean ± SD (n = 3). *p < 0.05.

Then we estimated the effect of BHX on the cell-cycle distribution. The A549 and MCF-7 cells which were treated with or without BHX were subjected to flow cytometry analysis. Data showed that compared with the control samples, BHX induced a G1 phase increased by 30% and a slight decrease in S phase (Fig. 5a,b). The above-mentioned data indicated that BHX arrested the cell cycle in the G0/G1 phase in a concentration-dependent manner.

### BHX prevents the transcription of β-catenin, but is not involved in GSK-3β-dependent phosphorylation of β-catenin

**Figure 6:**
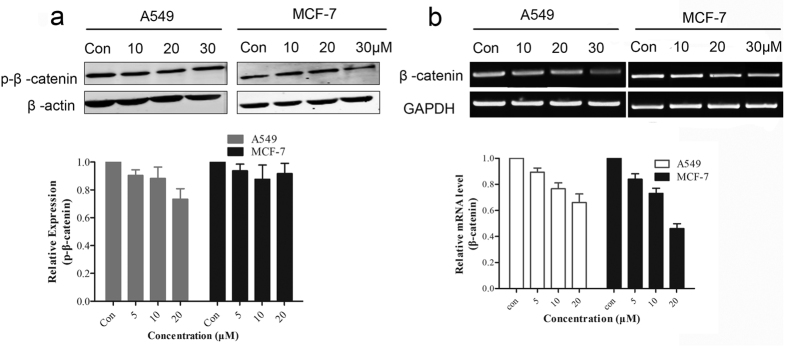
BHX decreased the β-catenin mRNA levels, but the p-β-catenin was barely modified. The expression of p-β-catenin in A549 and MCF-7 did not increase which compared with the control but BHX slightly decreased the p-β-catenin expression (**a**). mRNA expression of β-catenin in the cells treated with (0–20 μM) or without BHX in A549 and MCF-7 cells. GAPDH was used as internal control. BHX reduced the mRNA levels of β-catenin significantly (**b**). Results are mean ± SD (n = 3).

Accumulated β-catenin travels to the nucleus and activates the Wnt signaling pathway. The results have already showed that the expression of p-β-catenin did not increase which compared with the control but BHX slightly decreased the p-β-catenin expression (Fig. 6a). Therefore, we examined β-catenin at the transcriptional level by PCR, and results revealed that the mRNA level of β-catenin was decreased remarkably at 20 μM (Fig. 6b). Hence, BHX reduced the expression of β-catenin not by blocking the Wnt signaling pathway upstream but by reducing β-catenin mRNA levels, thereby reducing transcription and translation, and ultimately reducing protein expression. Thus, the total amount of β-catenin was reduced, and p-β-catenin levels also was lowered slightly.

## Discussion

The Wnt signaling pathway plays an indispensable role in normal development and is also important in oncogenesis. The malignant activation of the Wnt signaling pathway leads to the onset of multiple types of cancers^20^. Meanwhile, β-catenin is a key activator of the canonical Wnt signaling pathway^21^. The abnormal accumulation and nuclear translocation of β-catenin play an important role in all of the β-catenin mutations^22^. Therefore, restraining the expression of β-catenin and nuclear translocation can be a potential therapeutic strategy^23^.

In our study, BHX decreased the protein levels of β-catenin and the downstream target genes cyclinD1 and c-myc. BHX inhibited β-catenin expression by decreasing the molecule’s transcription. The PCR analysis showed that BHX induced a dose-dependent inhibitory effect on the β-catenin mRNA levels.

BHX effectively inhibited tumor cell proliferation *in vitro* and *in vivo*. The antitumor effects exerted a close relationship with the inhibition of the Wnt signaling pathway. Thus, we examined the Wnt signaling key factor β-catenin protein in the nucleus after being treated with BHX. In fact, the effects of BHX on the β-catenin protein expression levels were analyzed by immunofluorescence and Western blot, revealing that BHX decreased β-catenin levels in the cell nucleus. Down-regulating the nuclear β-catenin reduces the interaction of β-catenin and TCF/LEF, thereby suppressing the Wnt signaling pathway^24^. Herein, we also showed that the suppression of the nuclear translocation of β-catenin resulted in the down-regulated expression of cyclin D1 and c-myc, which were downstream oncogenes of the Wnt/β-catenin signaling pathway^25^,^26^. Cyclin D1 participates in the phase transitions of the cell cycle by phosphorylating the retinoblastoma protein. Cell-cycle regulation is pivotal in the control of cell proliferation and closely related to the Wnt signaling pathway^27^. After being treated with BHX, tumor cells were arrested in the G1 phase. Moreover, BHX induced cell-cycle arrest in a concentration-dependent manner.

As the indispensable mediator of the Wnt/β-catenin signaling pathway, β-catenin functions as a localization protein^28^. Membrane-localized β-catenin is isolated by the E-cadherin to maintain cell–cell adhesion. In addition, the classical Wnt signaling pathway causes the accumulation of β-catenin, and access to nucleus regulates target gene expression. In the absence of Wnt signaling, the level of β-catenin remained low through the degradation of cytoplasmic β-catenin, which is targeted for phosphorylation by CK1-α at the Ser45 site, followed by phosphorylation by GSK3-β at Ser33, Ser37, and Thr41^29^,^30^. This process then leads to ubiquitination. Thus, we detected β-catenin phosphorylation levels at the Ser45/Thr41 site in the cells treated with BHX. Interestingly, the phosphorylation levels were not elevated but also slightly lower than the control. Subsequently, we found that β-catenin mRNA levels decreased, implying that the synthesis of β-catenin was reduced. Consequently, β-catenin transcription and translation was down-regulated, decreasing β-catenin levels in the cytoplasm, and ultimately inhibiting the Wnt signaling pathway. Meanwhile, the Wnt signaling pathway is known to undergo crosstalk with other signaling pathways, such as the TGF-β, Notch, and MAPK signaling pathways^31^,^32^, which play important roles in the development of tumor cells. Thus, other mechanisms may be involved in the antitumor effect of BHX.

In conclusion, our findings provide evidence that BHX may inhibit tumor cell proliferation by attenuating the Wnt/β-catenin signaling pathway through nuclear β-catenin level reduction. Such mechanism was found to be accompanied by the down-regulation of cyclin D1 and c-myc, which is tightly connected to the development and prognosis of tumor cells. The mechanism probably involves the decrease in β-catenin transcription and translation. Cisplatin and any other first-line chemotherapy drug hold the disadvantage of strong toxicity and side effects; non-specificity of drug action is a reason for this phenomenon, thus limiting clinical application^33^. By contrast, the small-molecule inhibitor BHX targets tumor cells activated by the Wnt signaling pathway but only affects normal cells activated by the same pathway to a lower extent. Thus, BHX holds the potential to be developed to a safe therapeutic drug for Wnt-activated tumors.

## Materials and Methods

### Cell lines and culture conditions

Human tumor cell lines A549 (lung adenocarcinoma cell line) and MCF-7 (the breast cancer cell line) were acquired from the American Type Tissue Cell Culture Collection, whereas Beas-2b cells were frozen and stored in our laboratory. The A549 cells were cultured in RPMI 1640 (Gibco), and the MCF-7 cells were maintained in Dulbecco’s modified Eagle’s medium (DMEM, Gibco) supplemented with 10% fetal bovine serum (Gibco), 1% penicillin (100 IU/mL), and streptomycin (100 mg/mL) at 37 °C in an incubator under 5% CO_2_. The Beas-2b cells were cultured using LHC-8 medium (Gibco).

### Drug

BHX was dissolved in dimethyl sulfoxide (DMSO) at a final concentration of 40 mM, stored as a stock solution at −20 °C, and diluted in DMEM before using.

### Cell viability assay

The effect of BHX on cellular proliferation and viability was determined by MTT assay (R&D Systems, UK). The A549, MCF-7, and Beas-2b cells were seeded in 96-well plates at a density of 3.0 × 10^3^ cells/well in 100 μL of medium and allowed to attach overnight. The cells were then treated with BHX at increasing concentrations (0–80 μM) for 24, 48, and 72 h. Subsequently, the cells were incubated with the MTT reagent at a final concentration of 5 mg/ml for 4 h. Finally, the intracellular formazan crystals were solubilized with 150 μl DMSO. Absorbance was measured at 490 nm using an enzyme-linked immunosorbent assay plate reader, and the reduction in cell viability in different treatment groups was expressed as the percentage compared with BHX-treated and BHX-free control cells. All experiments were performed in triplicate.

### Colony-forming assay

Colony-forming assay was performed to evaluate the long-term drug efficiency. A549 and MCF-7 cells were seeded at 200 cells/well into 12-well plates with 1 ml DMEM and allowed to attach overnight. The cells were exposed to increasing concentrations (0–20 μM) of BHX for 72 h, followed by medium removal and incubation in fresh medium for 2 weeks at 37 °C. The cells were then fixed with 100% ethanol (4 °C, 20 min) and stained with 1% crystal violet (Sigma) for 15 min. Colonies that contained more than 50 cells were counted. Clonogenic capability was calculated as the ratio between treated counted clones and the corresponding number of clones control well. Each experiment was performed in triplicate.

### Cell-cycle analysis

Cell-cycle distribution was performed as described previously^34^. Briefly, cells were treated with BHX (0–20 μM) for 48 h. After treatment, attached cells were trypsinized, and then harvested and washed with PBS. Cells were centrifuged (1000 rpm, 5 min) and subsequently fixed with ice-cold 70% ethanol for 24 h. After centrifugation, cells were washed twice and then re-suspended in PBS containing propidium iodide (40 μg/ml), RNase A (100 μg/ml), and Triton X-100 (0.05%) and incubated at 37 °C for 15 min. Cell cycle was analyzed using a FACS flow cytometer.

### Western blot analysis

To determine the change of protein expression level, Western blot analysis was performed. Protein of the treated cells lysates was prepared by Bradford assay. Protein concentration was determined using Pierce BCA protein assay kit (Thermo Scientific). Equivalent amounts (50 μg protein/lane) of protein lysates were separated by 8% sodium dodecyl sulfate–polyacrylamide gel electrophoresis and transferred onto polyvinylidene membranes in a transfer tank (Bio-Rad Laboratories) using the submerged method. Membranes were blocked for 1 h at room temperature in 5% (w/v) fat-free milk powder in 1× TBS containing 1% Tween 20 and then incubated with the primary antibody (1:1000 dilution) with gentle agitation overnight at 4 °C. The primary antibodies were as follows: anti-β-catenin (1:1000 dilution, rabbit monoclonal antibody, Cell Signaling Technology), anti-β-actin (1:1000 dilution, mouse monoclonal antibody, Cell Signaling Technology), anti-cyclin D1 and anti-c-myc (1:1000 dilution, rabbit monoclonal antibody, Cell Signaling Technology), anti-Lamin B (1:1000 dilution, rabbit monoclonal antibody, Santa Cruz Biotechnology, Santa Cruz, CA), and anti-p-β-catenin (Ser^45^/Thr^41^, 1:200 dilution, rabbit monoclonal antibody, Santa Cruz Biotechnology, Santa Cruz, CA). Then, the membranes were washed three times with 0.1% Tween 20 in Tris HCL buffer and incubated with secondary goat anti-mouse-horseradish peroxidase (HRP) and goat anti-rabbit HRP antibodies (Bio-Rad) were used at 1:5000 for 1 h at room temperature. After final washing with 0.1% Tween 20 in TBS (three times for 10 min each), blots were developed using a luminescent image analyzer LAS-4000 (Fujifilm, Dielsdorf, Switzerland) and Multi Gauge software (Fujifilm Version 3.0).

### PCR analysis

A549 and MCF-7 cells were plated on a 6 cm dish at a density of 2 × 10^5^ per dish; each dish contained 3 ml medium, and the cells were allowed to attach overnight. The cells were then exposed to increasing concentrations (0–20 μM) of BHX for 72 h. Total RNA was extracted using TRIzol reagent (Life Technologies, USA) in accordance with the manufacturer’s instructions. Isolated RNA samples were quantified by a NanoDrop 3300 spectrophotometer (Thermo Scientific, NanoDrop Products, Wilmington, DE, USA). The isolated RNA (5 μg) was used to prepare complementary DNA using a RevertAid First Strand cDNA Synthesis Kit following manufacturer’s protocols (Thermo Scientific, USA). β-Catenin was amplified by a standard PCR protocol using 5′–ACAACTGTTTTGAAAATCCA–3′ as forward primer, 5′–CGAGTCATTGCATACTGTCC–3′ as reverse primer, and the prepared cDNA as a template. The internal control GAPDH adopted 5′–ACCACAGTCCATGCCATCAC–3′ as forward primer and 5′–TCCACCACCCTGTTGCTGTA–3′ as reverse primer. The reaction mixtures were heated at 95 °C for 10 min, followed by 30 cycles of 94 °C for 30 s, 58 °C for 30 s, 72 °C for 20 s, and a final extension at 72 °C for 5 min. Subsequently, PCR products were electrophoresed through 1.5% agarose gel and then subjected to a gel/fluorescence image analysis system for scanning.

### Immunofluorescence

For immunofluorescence studies, A549 and MCF-7 cells were plated on glass cover slips placed on 12-well plates. Cells were exposed to increasing concentrations (0–20 μM) of BHX for 72 h. Before staining, the cells were fixed with 4% paraformaldehyde (Sigma) for 10 min, permeabilized in 0.1% Triton X-100 for 15 min, and blocked in 3% BSA at room temperature for 1 h. Each previous step was accompanied by PBS wash twice. Cells were incubated with mouse monoclonal antibodies to β-catenin (1:200) overnight at 4 °C in a humid chamber and then incubated with goat anti-mouse Alexa-647 antibody (1:150) for 1 h at room temperature. Both primary and secondary antibodies were diluted in PBS. Representative images were captured by a Zeiss LSM510 Meta-confocal microscope.

### Tumor xenograft

Antitumor activity studies were performed on 8-week-old female BALB/c mice weighing about 20 g. Briefly, an A549 cell (2 × 10^7^/ml) suspension was injected into the right groin (150 μl growth medium) of each mouse. Tumor volume was calculated by the formula V = (*a* × *b*^2^)/2^35^, in which a is the length and b is the width. When the tumor volumes reached about 100 mm^3^ to 150 mm^3^, the mice were randomly divided into four groups, including BHX-treated, vehicle, and cisplatin-treated (six mice per group). The BHX-treated group received intraperitoneal injection at increasing dose levels of 50 and 100 mg/kg body weight for 3 weeks, consecutively. By contrast, the cisplatin-treated group received intraperitoneal injection at a dose of 4 mg/kg twice a week. Tumor volume was measured every 3 days, and body weight was measured daily for safety evaluation. All experimental protocols were approved by Animal Care Committee of Tianjin Medical University Cancer Institute and Hospital, China. And all the experiments were performed in accordance with relevant guidelines and regulations of Animal Care Committee of Tianjin Medical University Cancer Institute and Hospital.

### Statistical analysis

Data were expressed as mean ± SD. Statistical analyses were performed using the SPSS version 16.0 software. Multiple groups comparison was analyzed by one-way analysis of variance(ANOVA). Two group comparison was analyzed by unpaired two-tailed student’s t-test. A probability of P ≤ 0.05 was considered statistically significant.

## Additional Information

**How to cite this article**: Ding, F. *et al*. BHX Inhibits the Wnt Signaling Pathway by Suppressing β-catenin Transcription in the Nucleus. *Sci. Rep.* **6**, 38331; doi: 10.1038/srep38331 (2016).

**Publisher’s note:** Springer Nature remains neutral with regard to jurisdictional claims in published maps and institutional affiliations.
